# EGFR Amplification in Diffuse Glioma and Its Correlation to Language Tract Integrity

**DOI:** 10.3390/diagnostics15172266

**Published:** 2025-09-08

**Authors:** Alim Emre Basaran, Alonso Barrantes-Freer, Max Braune, Gordian Prasse, Paul-Philipp Jacobs, Johannes Wach, Martin Vychopen, Erdem Güresir, Tim Wende

**Affiliations:** 1 Department of Neurosurgery, University Hospital Leipzig, 04103 Leipzig, Germany; 2 Paul-Flechsig-Institute of Neuropathology, University Hospital Leipzig, 04103 Leipzig, Germany; 3 Institute of Neuroradiology, University Hospital Leipzig, 04103 Leipzig, Germany; 4 Department of Diagnostic and Interventional Radiology, University Hospital Leipzig, 04103 Leipzig, Germany

**Keywords:** diffuse glioma, EGFR amplification, diffusion tensor imaging, fractional anisotropy, language tract integrity

## Abstract

**Background**: The epidermal growth factor receptor (EGFR) is an important factor in the behavior of diffuse glioma, serving as a potential biomarker for tumor aggressiveness and a therapeutic target. Diffusion tensor imaging (DTI) provides insights into the microstructural integrity of brain tissues, allowing for detailed visualization of tumor-induced changes in white matter tracts. This imaging technique can complement molecular pathology by correlating imaging findings with molecular markers and genetic profiles, potentially enhancing the understanding of tumor behavior and aiding in the formulation of targeted therapeutic strategies. The present study aimed to investigate the molecular properties of diffuse glioma based on DTI sequences. **Methods**: A total of 27 patients with diffuse glioma (in accordance with the WHO 2021 classification) were investigated using preoperative DTI sequences. The study was conducted using the tractography software DSI Studio (Hou versions 2025.04.16). Following the preprocessing of the raw data, volumes of the arcuate fasciculus (AF), frontal aslant tract (FAT), inferior fronto-occipital fasciculus (IFOF), superior longitudinal fasciculus (SLF), and uncinate fasciculus (UF) were reconstructed, and fractional anisotropy (FA) was derived. Molecular pathological examination was conducted to assess the presence of EGFR amplifications. **Results**: The mean age of patients was 56 ± 13 years, with 33% females. EGFR amplification was observed in 8/27 (29.6%) of cases. Following correction for multiple comparisons, FA in the left AF (*p* = 0.025) and in the left FAT (*p* = 0.020) was found to be significantly lowered in EGFR amplified glioma. In the right language network, however, no statistically significant changes were observed. **Conclusions**: EGFR amplification may be associated with lower white matter integrity of left hemispheric language tracts, possibly impairing neurological function and impacting surgical outcomes. The underlying molecular and cellular mechanisms driving this association require further investigation.

## 1. Introduction

The treatment and perioperative neurosurgical management of diffuse gliomas represents a challenge in the field of neuro-oncology [1,2]. Among the biomarkers for diffuse gliomas, the epidermal growth factor receptor (EGFR) plays a clinically relevant role, particularly in terms of progression-free survival (PFS) and overall survival (OS) [3,4,5,6]. In the present study, the term EGFR is used to denote copy-number amplification as derived from methylation-specific copy-number profiles. However, the interrogation of EGFR promoter methylation is not included in this study. For the purposes of reference, the standardized, accepted form of the EGFR transcript designated for gene annotation is the National Center for Biotechnology Information (NCBI) Reference Sequence (NM_005228.5, Gene ID: 1956). Furthermore, we present the MGMT (O^6^-alkylguanine DNA alkyltransferase) promoter methylation status for the cohort (see Table 1). In addition to copy-number amplification, EGFRvIII is a characteristic molecular alteration in gliomas. It represents a constitutively active EGFR variant that frequently co-occurs with EGFR amplification [7]. Given its importance, advanced imaging techniques, such as diffusion tensor imaging (DTI), have become more interesting in preoperative mutation profiling and perioperative surgical planning for diffuse gliomas. DTI is a diffusion-MRI technique that models the directional (anisotropic) motion of water to infer the microstructural integrity and organization of white matter, enabling tract-specific assessment of fiber pathways. The primary DTI metric used in this study is fractional anisotropy (FA; range 0–1), which reflects the degree of directional coherence within a tract. Lower FA indicates microstructural disruption (e.g., edema, infiltration, fiber disorganization) [8,9]. Fractional anisotropy (FA) is a scalar (range 0–1) derived from the diffusion tensor that quantifies how directionally constrained water diffusion is within tissue: values near 1 indicate highly anisotropic, coherently oriented fiber architecture, whereas values near 0 indicate isotropic diffusion (e.g., cerebrospinal fluid or edema). As mentioned, lower FA is a sensitive marker of microstructural disruption, reflecting changes in axonal density, fiber organization/diameter, myelination, or free-water/edema [10,11]. These microstructural changes within the white matter tracts often precede abnormalities detectable by conventional imaging modalities and may therefore serve as potential imaging biomarkers [12,13,14]. Recent studies have shown that molecular alterations in diffuse gliomas, such as EGFR amplifications, IDH mutations, and 1p/19q co-deletions, not only impact prognosis and treatment strategies but also lead to characteristic changes in brain microstructure [12,15,16]. As outlined above, these alterations affect cell density, infiltration behavior, and the extent of peritumoral edema, all of which can be captured using DTI. DTI-derived metrics such as FA and mean diffusivity (MD) are particularly sensitive to such abnormalities and may provide non-invasive surrogate markers for underlying molecular and pathophysiological changes [13,14,17].

The aim of any neurosurgical procedure is to prevent the deterioration of neurological deficits and to maintain the quality of life (QoL) in patients. In order to maintain oncological and functional balance, as well as the associated QoL and preservation of neurological function in patients, it is essential to provide neurosurgeons with preoperative information on tumor location, infiltration, and preoperative neurological conditions, as well as molecular markers if possible [18,19]. DTI plays a crucial role in the preoperative mapping of white matter tracts, which enables more precise resection while preserving important brain functions, while simultaneously minimizing post-operative neurological deficits, and can be operationalized as an automated, tract-specific assessment to inform clinical decision-making. This allows better preoperative planning and consequently better perioperative management [20,21,22]. DTI enables the quantification of white matter tract integrity, providing insights into the infiltrative nature of tumors and their impact on brain connectivity [23]. The ability to correlate DTI-derived measurements with molecular alterations, such as EGFR mutation profiles, offers valuable preoperative information, facilitating the optimization of neurosurgical planning and personalized treatment approaches [24]. Previous studies have demonstrated that preoperative DTI can discriminate between IDH-mutant and IDH-wildtype astrocytomas [12,25,26,27]. However, the specific relationship between EGFR mutations and changes in DTI parameters remains unclear.

Moreover, correlating EGFR amplification with tract-specific DTI parameters has direct implications for functional neurosurgery. Language pathways such as the AF (arcuate fasciculus) and FAT (frontal aslant tract) play a central role in preserving speech and higher-order cognitive functions. Identifying microstructural compromise in these tracts may help stratify patients at greater risk for postoperative deficits, influence the choice between awake and asleep craniotomies, and guide the extent of resection in eloquent regions. This convergence of radiogenomics and functional imaging could thus provide a framework for tailoring both oncological and functional strategies, optimizing the delicate balance between maximal resection and preservation of neurological function.

## 2. Materials and Methods

### 2.1. Participants

This study was approved by the ethics committee of Leipzig University (217/24-ekm approved on 25 February 2025) and was performed in accordance with the relevant guidelines and regulations. Informed consent was waived by the ethics committee due to the retrospective, pseudonymized nature of the study. This study was conducted at the Department of Neurosurgery, University Hospital Leipzig. Patients who underwent surgery between 2021 and 2023 were included in the study. All patients were neuropathologically diagnosed with astrocytoma (grade 2–4) or glioblastoma (grade 4) according to the 2021 WHO classification. The inclusion criteria for the study were: (1) a gross total resection (GTR) or subtotal resection (STR). (2) A neuropathological confirmed diagnosis of glioma, (3) whole genome DNA methylation analysis of the tumor sample, (4) preoperative MRI scans with DTI sequences, and (5) no other intracranial pathologies und malignancies that could confound imaging or molecular analyses. Additional consideration was given to the timing of imaging acquisition, ensuring that all preoperative scans were performed within a standardized window prior to surgery to reduce variability. Furthermore, only patients with complete clinical documentation and postoperative follow-up data were included, allowing for more comprehensive correlation of imaging and molecular findings. This careful selection aimed to enhance the reliability of our results and minimize potential confounding factors. Patients with incomplete imaging data, prior cranial surgery, or insufficient tissue for molecular analysis were excluded.

### 2.2. Molecular Pathology

Genome-wide DNA methylation analysis was performed using the Infinium Methylation EPIC (850k) Beadchip (Illumina, San Diego, CA, USA) for all tumor samples using standard techniques. This platform allows high-resolution, genome-wide profiling of CpG methylation sites, enabling both diagnostic refinement and molecular subclassification of gliomas in accordance with the 2021 WHO classification. The methylation profile of each tumor was compared to previously defined methylation classes using the publicly available classifier of the German Center for Cancer Research (DKFZ) available online: www.molecularneuropathology.org (accessed on 11 June 2025) [28]. This classification approach ensures a robust and standardized assessment of glioma subtypes, improving diagnostic accuracy and reproducibility across centers. The log^2^ value of EGFR was obtained from copy number variation (CNV) profiles derived from the methylation data, where values above the defined threshold were considered indicative of EGFR amplification [29]. This integrated methylation and CNV approach not only allows for the identification of epigenetic subtypes but also provides a reliable, genome-wide estimate of key genetic alterations, making it a valuable tool for precision diagnostics in diffuse gliomas. Molecular pathological analyses (DNA methylation array processing, classifier-based tumor classification, and CNV determination for EGFR amplification) were performed independently of clinical variables, imaging findings (including tractography and fractional anisotropy metrics), and patient outcomes. This was only carried out for the tumor sample labelled with the suspected diagnosis of diffuse glioma, for which no additional clinical or imaging information was provided. In this retrospective cohort, EGFRvIII was not systematically genotyped. EGFR amplification was determined from methylation-derived CNV profiles. Values above the predefined threshold were considered amplified, as reported in Table 1. However, it should be noted that the assessment of EGFR promoter methylation was not included in the retrospective cohort study.

### 2.3. Imaging

Preoperative workup included 3 T MRI (Ingenia, Philips, Eindhoven, The Netherlands). The DTI acquisition consisted of single-shot epi-planar imaging (EPI) sequences using the following parameters: number of diffusion directions = 32, repetition time = 7010 ms, echo time = 102 ms, b-value = 1000 s/m^2^, number of b = 0 volumes: 1, field of view = 222 × 222 mm^2^, voxel size = 1.98 × 1.98 × 2.7 mm^3^, number of excitations = 1, gap between slices = 0, and acquisition time = 330 s. Preprocessing was carried out using FSL topup and eddy in DSI Studio (https://dsi-studio.labsolver.org/, Hou versions 2025.04.16). Imaging and preprocessing results were checked visually for quality. Automated segmentation of white matter tracts was performed in DSI Studio [30] with pruning and automatic check-ending [31]. Tract segmentations underwent an additional visual quality control step to identify potential errors related to the altered brain anatomy, such as false-positive segmentation of edema, tumor, or adjacent fiber bundles, and false-negative segmentation of anatomically distinct parts of the respective tracts.

**Figure 1:**
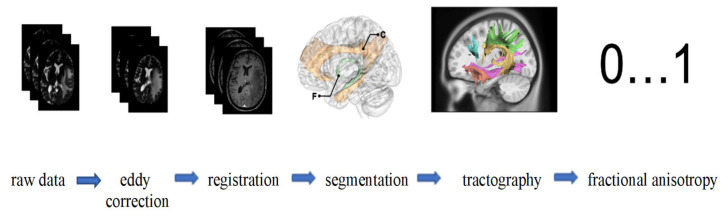
Starting with raw MRI data, eddy correction is applied to address motion and eddy current distortions. The images are then aligned with a standard space through registration. Following this, segmentation isolates specific brain regions [31]. (Exemplaric: C, cingulum; F, fornix). Tractography is subsequently performed to map white matter tracts, visualizing fiber pathways. Finally, fractional anisotropy (FA) values, ranging from 0 to 1, are computed to quantify the directional coherence of water diffusion, which provides insights into white matter integrity.

FA values were produced tract-wise using the automatically generated segmentation maps. Figure 1 illustrates the entire processing workflow from raw data acquisition through preprocessing, registration, segmentation, and tractography to the extraction of FA values, highlighting the stepwise approach used to ensure high-quality and reproducible diffusion metrics. This comprehensive pipeline of preprocessing, multi-step registration, and automated tract segmentation was specifically designed to address the challenges of imaging patients with diffuse gliomas, where brain anatomy is frequently distorted. The combination of advanced denoising, artifact correction, and manual quality control increases the robustness of the derived FA values, particularly in peritumoral regions where diffusion properties are often most affected. These methodological safeguards enhance the validity of the tract-specific analyses and ensure that the resulting diffusion metrics can be interpreted with confidence in the context of radiogenomic correlations.

### 2.4. Data Analysis

Statistical analysis was performed using SPSS Statistics version 29 (IBM, Armonk, NY, USA). Independent-sample *t*-tests were used to compare fractional anisotropy (FA) values between EGFR-amplified and non-amplified tumors. To reduce the risk of false-positive results from multiple comparisons, *p*-values were adjusted using the Benjamini–Hochberg method with a false discovery rate of 0.05. A *p*-value below 0.05 was considered statistically significant. Boxplots were created using Seaborn (version 0.11.1) and Matplotlib (version 3.10.6) [32]. Our prespecified primary endpoint was FA, analyzed tract-wise and separately by hemisphere for language pathways: arcuate fasciculus (AF), frontal aslant tract (FAT), inferior fronto-occipital fasciculus (IFOF), superior longitudinal fasciculus II (SLF-II), uncinated fasciculus (UF). Multiple comparisons were controlled using Benjamini-Hochberg FDR (*q* = 0.05). FA was summarized tract-wise using tract segmentations. Voxelwise skeleton-based tests or distance-to-lesion modelling were not performed. Consequently, the presence of local tumor and edema effects along a given tract may contribute to variations in FA, which cannot be fully disentangled from molecular influences in this study.

## 3. Results

### 3.1. Patient Cohort

A total of 27 patients were included in the study. According to RANO resection guidelines, 20 patients underwent resection based on Grade 1, and 7 patients underwent resection based on Grade 2 [33]. A total of 27 patients were examined, of whom 9 (33.3%) were male and 18 (66.7%) were female. An EGFR amplification was detected in 8 patients (30%), whereas no amplification was present in 19 patients (70%). Within these categories, EGFR amplification was observed in 1/27 (3.7%) IDH-mutant and 7/27 (25.9%) IDH-wildtype tumors, whereas EGFR was not amplified in 8/27 (29.6%) and 11/27 (40.7%), respectively; with respect to MGMT, EGFR was amplified in 7/27 (25.9%) unmethylated and 1/27 (3.7%) methylated cases, and was absent in 13/27 (48.1%) and 6/27 (22.2%) cases, respectively.

**Figure 2:**
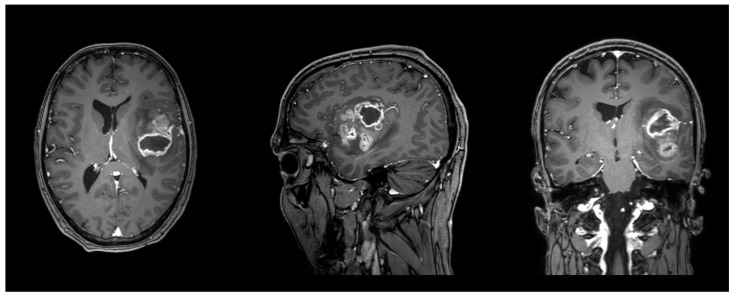
Illustrative case of a male patient in his forties with a left frontotemporal glioblastoma, WHO grade 4, IDH wildtype, MGMT promoter not methylated, EGFR amplified. FA in the left AF was 0.42, and 0.38 in the left FAT. T1 sequence with contrast enhancement. Planes are in radiological orientation, from left to right: axial, sagittal, coronal.

One patient was lost to follow-up. With respect to tumor location, the majority of lesions involved the left hemisphere (*n* = 19), most frequently affecting the temporal (*n* = 9) and frontal lobes (*n* = 3), whereas right hemispheric tumors (*n* = 18) were predominantly located in the frontal (*n* = 6) and temporal lobes (*n* = 5). One case demonstrated corpus callosum involvement. With regard to the issue of laterality. The present investigation is exclusively focused on the issue of lesion laterality. The distribution of responses is as follows: In the present study, 27 patients were analyzed and the results indicated that nine of these patients (33.3%) were classified as left-only, eight patients (29.6%) were classified as right-only, and 10 patients (37.0%) were classified as bilateral. The lobar distribution data presented in Table 1 are non-exclusive, meaning that a tumor may involve multiple lobes and/or both hemispheres. Detailed demographic data, tumor characteristics, and neuroanatomical locations are summarized in Table 1. An illustrative case can be found in Figure 2.

**Table 1:** Patient characteristics.

**Patients (*n*)**	27	
**Age**	56 ± 12.9	
**Sex**		
Male	9/27 (33.3%)	
Female	18/27 (66.7%)	
**WHO Grade**		
2	1/27 (3.8%)	
3	4/27 (15.4%)	
4	22/27 (81.5%)	
**Lesion laterality (exclusive)**		
Left only	9/27 (33.3%)	
Right only	8/27 (29.6%)	
Bilateral	10/27 (37.0%)	
Neuroanatomical Tumor Spread (non-exclusive counts *)		
Left hemisphere	Σ19	
Left frontal	3	
Left temporal	9	
Left parietal	3	
Left occipital	0	
Left cerebellum	0	
Left central	4	
Left thalamus	0	
Right hemisphere	Σ18	
Right frontal	6	
Right temporal	5	
Right parietal	2	
Right occipital	2	
Right cerebellum	0	
Right central	1	
Right thalamus	2	
Corpus callosum	1	
**EGFR amplified**		
No	19 (70.4%)	
Yes	8 (29.6%)	
**IDH status**	EGFR amplified	EGFR not amplified
Mutated	1/27 (3.7%)	8/27 (29.6%)
Wildtype	7/27 (25.9%)	11/27 (40.7%)
**MGMT methylation status**	EGFR amplified	EGFR not amplified
Unmethylated	7/27 (25.9%)	13/27 (48.1%)
Methylated	1/27 (3.7%)	6/27 (22.2%)

* Non-exclusive counts: a tumor may involve multiple regions and/or both hemispheres; totals can exceed *n* = 27. Exclusive lesion laterality (Left-only/Right-only/Bilateral) is reported above.

### 3.2. White Matter Integrity

**Figure 3:**
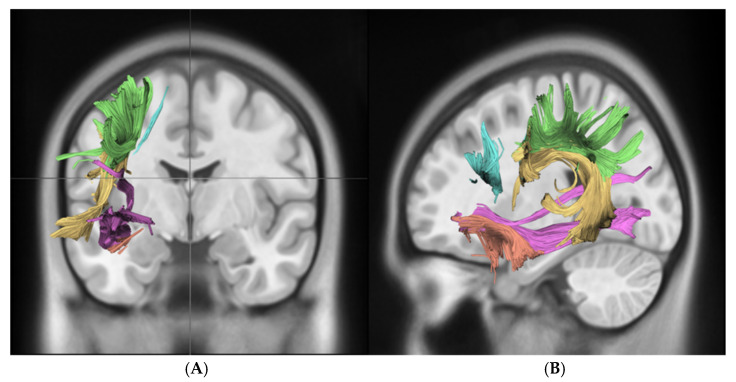
This figure demonstrates key white matter tracts in the brain using DTI, shown in coronal (**A**) and sagittal (**B**) views. Color-coded tracts include the SLF in green, AF in yellow, FAT in blue, UF in orange, and IFOF in violet. These images highlight the spatial orientation and connectivity of these tracts across different brain regions. **Abbreviations:** superior longitudinal fasciculus (**SLF, green**), arcuate fasciculus (**AF, yellow**), frontal aslant tract (**FAT, blue**), uncinate fasciculus (**UF, orange**), inferior fronto-occipital fasciculus (**IFOF, purple**).

**Figure 4:**
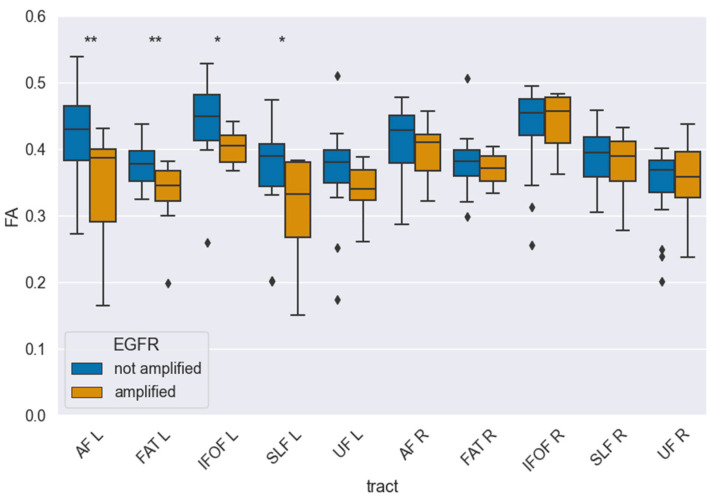
Tract-wise fractional anisotropy (FA) stratified by EGFR amplification (amplified vs. not amplified) for language-related pathways (AF, FAT, IFOF, SLF-II, UF) in each hemisphere. Boxplots depict median, interquartile range, and outliers. After FDR correction, FA was significantly lower in EGFR-amplified tumors in AF-L (*p_corr_* = 0.025) and FAT-L (*p_corr_* = 0.020), while no tract in the right hemisphere reached significance in multiple comparisons. One asterisk (*) denotes univariate significance, two asterisks (**) denote significance in multiple comparisons.

For the statistical analysis of EGFR amplification, we performed a hemisphere-specific analysis, separating results for the right (R) and left (L) hemispheres. The correlation between fractional anisotropy (FA) and EGFR amplification was calculated for the following language-related and association white matter tracts: the arcuate fasciculus (AF), frontal aslant tract (FAT), inferior fronto-occipital fasciculus (IFOF), superior longitudinal fasciculus (SLF), and uncinate fasciculus (UF). A significant reduction in FA values was observed in the left AF (0.34 ± 0.04 vs. 0.41 ± 0.02, *p_corr_* = 0.025) and the left FAT (0.33 ± 0.02 vs. 0.38 ± 0.01, *p_corr_* = 0.020) in EGFR-amplified tumors compared to non-amplified cases. Importantly, no tract in the right hemisphere reached significance after multiple comparisons correction, indicating a localized, left-hemispheric pattern rather than a diffuse whole-brain effect. In contrast, no statistically significant FA changes were detected in the analyzed tracts of the right hemisphere. Median FA values for each tract stratified by EGFR amplification status are presented in Table 2, while Figure 3 illustrates the tractography-based reconstruction and spatial orientation of these pathways. In addition, Figure 4 visualizes the tract-wise FA distributions for EGFR-amplified vs. non-amplified diffuse gliomas and highlights the group differences, which are confined to the left-hemispheric AF and FAT. After correction for multiple comparisons, the findings indicate that lower FA values in the left AF and FAT are significantly associated with EGFR amplification, suggesting selective vulnerability of these left-hemispheric language tracts in diffuse gliomas with this molecular alteration.

## 4. Discussion

The aim of this study was to investigate the association between EGFR amplification and white matter tract integrity in diffuse glioma. To date, no investigation has examined the relationship between EGFR amplification, a copy-number alteration, and tract-specific, hemisphere-aware DTI metrics within language pathways. In this study, we found that EGFR amplification may be associated with lower FA values in the left AF and the left FAT in patients with diffuse glioma. Our hemisphere-aware, tract-specific analysis shows that EGFR amplification is associated with selective FA reduction in left-hemispheric language pathways (AF, FAT). Amplification-driven proliferation/invasion and peritumoral edema likely disrupt axonal coherence and myelin organization, thereby lowering FA [34]. Given the predominance of left-hemispheric language dominance in adults, these tracts may be preferentially vulnerable in EGFR-amplified tumors, aligning a molecular alteration with a network-level microstructural signature.

Previous studies have shown that FA is lower in the presence of both low-grade gliomas (LGG) and high-grade gliomas (HGG) compared to unaffected white matter tracts [35]. Yuan et al. developed a model using DTI-derived FA and mean diffusivity (MD) maps, combined with structural MRI, to predict IDH mutation status in gliomas. Their study highlighted the diagnostic value of DTI features, including FA, in predicting IDH mutation status, a key genetic marker in gliomas [12]. Although diffuse glioma is often described as a network-level brain disease, the tract-wise FA findings in our cohort point to selective, left-hemispheric changes associated with EGFR amplification (AF and FAT), rather than diffuse whole-brain involvement [29]. Therefore, FA reduction in the presence of EGFR amplification indicates a mechanism of accelerated tumor infiltration. This hypothesis would be consistent with the findings of previous studies on diffuse glioma, which involved the whole brain [36,37,38]. Nevertheless, we identified statistically significant EGFR amplifications only in the left hemisphere. This implies that eloquent areas in the left hemisphere might be more susceptible to EGFR amplifications.

The results of our present study indicate that EGFR amplification of diffuse gliomas may lead to lower FA values in the white matter tracts, specifically the left AF and the left FAT. Several potential explanations could account for these findings. Changes in cellular density in and around the tumor could alter diffusion properties, resulting in decreased FA. This has been observed in previous studies comparing IDH-mutant and IDH-wildtype gliomas. Additionally, FA values are correlated with the proliferative potential of gliomas, and different genetic profiles (e.g., IDH-mutant vs. IDH-wildtype) may lead to varying degrees of tumor proliferation, which in turn affects FA values [12]. Previous studies showed that EGFR amplification correlates significantly with peritumoral edema in glioblastoma [39,40]. The EGFR amplification results in overexpression of EGFR proteins in glioma cells. This results in tumor proliferation, angiogenesis, and invasion via RAS and PI3K/AKT signaling pathways. Consequently, vascular permeability increases, and this is followed by edema formation [3]. These mechanisms may also have implications for functional outcomes.

Disruption of the left arcuate fascicle and frontal aslant tract is strongly associated with deficits in speech production and language processing. Thus, our findings suggest that EGFR-amplified tumors might pose a higher risk for postoperative functional impairments in eloquent regions. This highlights the potential role of preoperative tractography not only in surgical planning but also in risk stratification for functional outcomes. This functional perspective is of particular clinical interest, as preoperative detection of language pathway compromise could influence the choice of surgical strategies (e.g., awake craniotomy, intraoperative mapping) and help balance maximal safe resection with preservation of critical functions. Incorporating tract-specific FA alterations into multidisciplinary decision-making may thus translate into better perioperative neurological outcomes.

Other findings suggest that genetic alterations can vary between contrast-enhancing and non-enhancing regions of gliomas. This heterogeneity may contribute to differences in FA values across the tumor and surrounding white matter. Additionally, in IDH-wildtype gliomas, there is evidence of early evolutionary divergence in cells at the tumor periphery compared to those at the center, potentially resulting in distinct genetic profiles and, consequently, varying effects on white matter integrity and FA values [41]. Several studies have shown that EGFR amplification is associated with increased tumor aggressiveness and potentially poorer outcomes, underscoring the importance of identifying molecular mutations preoperatively to guide treatment decisions and optimize patient care [42,43]. From a translational perspective, integrating radiogenomic markers such as FA reduction with molecular profiling could enable earlier and more accurate preoperative identification of aggressive glioma subtypes. This may allow for expedited initiation of targeted therapies or inclusion in clinical trials, particularly for patients with poor prognostic markers such as EGFR amplification. Such integration of imaging and molecular diagnostics is an evolving paradigm in neuro-oncology. Radiogenomics offers the opportunity to obtain actionable biological information preoperatively, which could reduce the time to treatment initiation and support enrollment in precision medicine trials, especially for patients with rapidly progressing disease. This functional perspective is of particular clinical interest, as preoperative detection of language pathway compromise could influence the choice of surgical strategies (e.g., awake craniotomy, intraoperative mapping) and help balance maximal safe resection with preservation of critical functions. Incorporating tract-specific FA alterations into multidisciplinary decision-making may thus translate into better perioperative neurological outcomes.

Preoperative detection of EGFR amplifications through non-invasive methods, such as DTI, could significantly impact surgical planning and improve patient outcomes in diffuse gliomas. Identifying EGFR amplification, which is associated with poorer outcomes, may prompt neurosurgeons to pursue more extensive resections. This is crucial, as studies have demonstrated that maximal resection is associated with improved progression-free survival and overall survival in glioma patients [33,44]. Furthermore, preoperative knowledge of EGFR status can inform decisions about adjuvant therapy and support more personalized treatment approaches. These personalized approaches include intraventricular application of targeted drugs, with EGFR being one of the most promising targets recently [45]. Thus, patients have to be screened for eligibility for these drugs before surgery, where a tappable reservoir is implanted, and treatment begins after molecular diagnosis. To the best of our knowledge, this is the first study that presents evidence, though retrospective, for preoperative assessment of EGFR status in glioma. Our findings may thus accelerate treatment decisions in precision oncology in the future.

Adjuvant therapy should be started promptly after surgery; however, the effort required for molecular profiling can result in delays in its initiation [46,47]. Blobner et al. reported that the median time for completing molecular profiling was 34 ± 15 days (range: 8–91 days), which could potentially delay the start of adjuvant therapy and negatively impact patient outcomes [48]. This is the first study showing that EGFR mutations can cause specific changes in DTI, especially in FA values. These findings could offer a new perspective on the preoperative identification of molecular mutation profiles, which can enhance perioperative management and lead to better outcomes for patients with diffuse gliomas. While there are variations in the exact cutoff point, evidence consistently suggests that delays beyond 6–8 weeks in starting adjuvant therapy are associated with poorer outcomes for glioblastoma patients [49,50,51]. Lower FA may therefore specifically trigger molecular profiling in a multidisciplinary approach. Additionally, our findings support the concept of a combined imaging–molecular workflow in glioma management. Rapid preoperative imaging biomarkers such as FA could serve as an early surrogate for aggressive molecular features, enabling teams to plan more radical resections, earlier initiation of chemoradiotherapy, and consideration for intraventricular or systemic targeted therapies. By bridging the gap between radiology and molecular pathology, such workflows could redefine the preoperative management of diffuse gliomas. Integrating these approaches into tumor boards and treatment pathways may accelerate clinical decision-making, particularly for high-risk patients. In routine care, tract-specific DTI can be operationalized through an automated pipeline that generates two types of data. Firstly, tract segmentations for language pathways (AF, FAT, IFOF, SLF-II, UF). Secondly, per-tract FA summaries. Prior to surgery, these outputs can be reviewed at the multidisciplinary tumour board in conjunction with molecular data (e.g., EGFR amplification) to stratify language risk and plan the surgical strategy. In patients with left-hemispheric, language-eloquent tumours, reduced FA in the left AF/FAT cortex may necessitate consideration of awake mapping, more conservative resection margins near eloquent fibers, or tailored counselling regarding potential deficits. Tractography can be exported to neuronavigation systems to support trajectory planning. After the operation, the repetition of DTI when clinically indicated enables longitudinal assessment of white-matter integrity relative to the contralateral homotopic tract and the patient’s own baseline. This aids rehabilitation and follow-up decisions. There is a risk of confounding factors due to tumor location. Gliomas often develop and grow along white matter pathways, and tumor-induced axonal injury can reduce diffusion anisotropy regardless of genotype. Recent experimental data suggest that early gliomas tend to expand into white matter, causing axonal injury and Wallerian-like degeneration, which could lower FA in affected areas and even drive tumor progression [52]. Within our tract-wise, hemisphere-aware framework, we therefore interpret EGFR-associated reductions in FA as a network-level signal that may be partly driven by spatial co-localization with language pathways (AF, FAT). We acknowledge that distance-to-tumor and voxel-wise analyses were not performed in this retrospective cohort, and this must be considered when interpreting the results.

While DTI/FA is sensitive to microstructural disruption, its capabilities are limited by crossing-fiber architecture and peritumoral edema/partial-volume, which can reduce anisotropy without distinguishing the underlying cause. The neurite orientation dispersion and density imaging (NODDI) model is capable of visualizing multiple tissue compartments and provides metrics such as free-water fraction. These metrics facilitate the differentiation of edema-related free water from axonal/neurite loss and the quantification of fiber dispersion in crossing regions. In a prospective setting with multi-shell diffusion acquisition, the combination of tract-specific FA with NODDI metrics might have the potential to evaluate whether left-hemispheric FA reductions (AF, FAT) are indicative of reduced neurite density, increased dispersion, or free-water effects. This approach has the potential to enhance biological specificity beyond that achieved by FA alone [53,54]. It is important to note that the EGFRvIII variant, a characteristic feature of gliomas, was not included in the analysis of this dataset. It is imperative that future studies evaluate whether the status of EGFRvIII modulates tract-specific diffusion changes independently of, or synergistically with, amplification, given that it has been observed to accompany EGFR amplification.

It is evident that the applications of diffusion tensor imaging and tract-specific FA extend beyond gliomas, encompassing other pathologies characterized by white-matter involvement. In Alzheimer’s disease (AD), the presence of atrophy-associated reductions (AARs) is frequently observed in long-association pathways and commissural fibers, indicative of network degeneration [55,56]. In multiple sclerosis (MS), the decrease in FA reflects demyelination and axonal loss both within lesions and in normal-appearing white matter [57,58]. In the context of HIV-associated neurocognitive disorder, there have been reports of diffuse FA reductions in the callosal and frontostriatal pathway [59,60]. These illustrations demonstrate the translational potential of automated, tract-based FA summaries for differential diagnosis and longitudinal monitoring. However, it should be noted that FA is not disease-specific, and its interpretation should be integrated with disease-specific markers (e.g., amyloid/tau in AD, inflammatory activity in MS, viral control in HIV).

The study also has several limitations. The spatial resolution of DTI (1–2 mm) may limit the detection of subtle white matter changes, potentially obscuring finer details linked to EGFR amplification [61,62]. The sample size of 27 patients, while providing valuable insights, limits the generalizability of the findings. Nonetheless, a significant correlation between EGFR amplification and white matter changes was observed, supporting the study’s hypothesis. Additionally, DTI captures macroscopic white matter changes but does not reveal cellular processes underlying EGFR amplifications. Furthermore, gliomas are known to occur more frequently along white matter tracts, which may contribute to the observed results and represent a potential limitation in interpreting the findings [63].

We also did not perform whole-brain voxel-wise text or global white-matter FA summaries. Therefore, subtle diffuse changes outside the investigated tracts cannot be excluded and merit assessment in larger cohorts. Although molecular pathology was blinded to detailed clinical and imaging data, the provision of a generic suspected diagnosis for sample processing could theoretically introduce minimal bias. However, EGFR amplification calls were generated from a standardized methylation/CNV pipeline using uniform threshold and quality control, mitigating subjective influence. It is further possible that by the retrospective nature of the presented data, eloquent tumor location is overrepresented due to earlier symptom onset and wider preoperative diagnostic workup. This effect is not uncommon and should be considered, especially due to the significant differences in the left AF and the left FAT, which might directly correlate to language deficits. However, in the absence of EGFR amplification, FA was similar between both hemispheres. Prospective studies would thus have to take into account the time between tumor genesis and diagnosis in diffuse glioma. This retrospective study utilized single-shell DTI, precluding multi-compartment modelling (e.g., NODDI). The development of future multi-shell protocols holds significant potential for neurite density, dispersion, and free-water estimation, with demonstrated reproducibility in clinical time acquisitions [53,64].

Future studies should also explore advanced diffusion models, such as neurite orientation dispersion and density imaging (NODDI) or free-water corrected DTI, which may provide greater sensitivity to microstructural changes. Combining these techniques with multi-omics profiling could refine the radio genomic characterization of gliomas and potentially reveal new therapeutic targets. These limitations underscore the need for prospective multi-center studies with larger cohorts and advanced imaging combined with detailed molecular profiling.

## 5. Conclusions

In conclusion, these results suggest that EGFR amplification in diffuse gliomas might be associated with significant reductions of fractional anisotropy (FA) values, particularly in the left hemisphere’s arcuate fascicle and frontal aslant tract. This selective microstructural disruption highlights the potential impact of EGFR-driven tumor biology on eloquent language networks, which may contribute to functional impairment and influence postoperative outcomes. The ability to detect such molecular alterations non-invasively using DTI offers valuable preoperative insights, enhancing both neurosurgical planning and neuropathological assessment. These findings emphasize the promise of radiogenomic imaging biomarkers in everyday clinical workflows, enabling earlier identification of aggressive tumor subtypes, personalized operative strategies, and better integration of targeted therapies. Ultimately, prospective, multi-center studies with larger cohorts, advanced diffusion models, and integrated molecular profiling are warranted to validate these findings and explore their implications for functional preservation and precision oncology, aiming to improve outcomes in this challenging patient population. At the clinical level, the incorporation of tract-specific DTI assessments delivered via automated pipelines into preoperative planning and postoperative follow-up has the potential to complement molecular profiling (e.g., EGFR) and support language-preserving surgical strategies. Prospective studies should integrate tract-specific FA with NODDI (NDI/ODI/free water fraction) to disentangle the effects of edema/free water from true axonal/neurite disruption in language pathways, thereby refining the EGFR-related microstructural signature. Beyond the field of neuro-oncology, tract-specific FA pipelines have the potential to be adapted to other diseases involving white matter (e.g., AD, MS, HIV-associated neurocognitive disorder), where longitudinal FA may complement disease-specific biomarkers and clinical staging.

## Figures and Tables

**Table 2:** Tract-specific differences by EGFR amplification.

Tract	FA Mean ± SEM (EGFR Amplified)	FA Mean ± SEM (EGFR Not Amplified)	*p*-Value (Unadjusted)	*p*-Value (Corrected)
AF-L	0.34 ± 0.04	0.41 ± 0.02	0.030	*0.025*
FAT-L	0.33 ± 0.02	0.38 ± 0.01	0.008	*0.020*
IFOF-L	0.40 ± 0.01	0.44 ± 0.01	0.044	0.055
SLF-II-L	0.33 ± 0.03	0.39 ± 0.01	0.037	0.055
UF-L	0.34 ± 0.01	0.37 ± 0.02	0.127	0.127
AF-R	0.40 ± 0.02	0.42 ± 0.01	0.206	0.392
FAT-R	0.37 ± 0.01	0.38 ± 0.01	0.319	0.392
IFOF-R	0.44 ± 0.02	0.43 ± 0.02	0.392	0.392
SLF-II-R	0.39 ± 0.02	0.39 ± 0.01	0.294	0.392
UF-R	0.36 ± 0.02	0.35 ± 0.01	0.333	0.392

Abbreviations: arcuate fasciculus (AF); frontal aslant tract (FAT); inferior fronto-occipital fasciculus (IFOF); superior longitudinal fasciculus (SLF); uncinate fasciculus (UF); left (L); right (R); fractional anisotropy (FA).

## Data Availability

The datasets generated and/or analyzed during the current study are not publicly available due to patient privacy and ethical restrictions but are available from the corresponding author on reasonable request. Access may require a date use agreement and approval by the institutional ethics committee.

## Abbreviations

Arcuate fasciculus (AF); frontal aslant tract (FAT); inferior fronto-occipital fasciculus (IFOF); superior longitudinal fasciculus (SLF); uncinate fasciculus (UF); fractional anisotropy (FA); diffusion tensor imaging (DTI); epi-planar imaging (EPI); quality of life (QoL); gross total resection (GTR); subtotal resection (STR); Response Assessment in Neuro-Oncology (RANO); World Health Organization (WHO); isocitrate dehydrogenase (IDH); O6-methylguanine-DNA methyltransferase (MGMT); copy-number variation (CNV); German Cancer Research Center (DKFZ); overall survival (OS); progression-free survival (PFS); Alzheimer’s Disease (AD); Multiple sclerosis (MS); neurite orientation dispersion and density imaging (NODDI); O6-alkylguanine DNA alkyltransferase (MGMT); copy number variation (CNV).
